# Potential pitfalls in diagnosis of immunotherapy-induced hypothalamic–pituitary–adrenal axis abnormalities: a clinical case

**DOI:** 10.1530/EO-21-0023

**Published:** 2022-06-28

**Authors:** Yixi Bi, Safwaan Adam, Viktoria Chatzimavridou, Paul Lorigan, Yinglai Huang

**Affiliations:** 1 The Christie’s NHS Foundation Trust, Manchester, UK; 2 Division of Breast and Endocrine Surgery, Borås Hospital, Borås, Sweden

**Keywords:** short synacthen test, SST, hypophysitis, immunotherapy, ACTH deficiency, cortisol, immune checkpoint inhibitor, assay

## Abstract

**Summary:**

Short synacthen tests (SST) are frequently used for assessing adrenocorticotropin hormone (ACTH) deficiency. In this study, we present the case of a 53-year-old man receiving immunotherapy for metastatic melanoma, who subsequently developed immune checkpoint inhibitor (ICI)-induced hypothyroidism and was investigated for the presence of ICI-induced hypocortisolaemia on different occasions. Despite two reassuring SSTs, he subsequently developed clinical and biochemical evidence of ACTH deficiency. The ACTH on local measurement was not conclusive in keeping with ICI-related ACTH deficiency but when repeated using an alternative assay confirmed the diagnosis. The case illustrates the evolution of ACTH deficiency and exposes the potential pitfalls of screening strategies. Two important lessons may be gleaned from this case: (i) SSTs can be normal in early cases of secondary adrenal insufficiency, for example, hypophysitis due to adrenal reserve and (ii) when there is mismatch between the clinical and biochemical presentation, the ACTH should be repeated using a different assay.

**Learning points:**

- Short synacthen tests, useful for ruling out adrenalitis and primary adrenal failure, may be normal in early adrenocorticotrophic hormone deficiency and secondary adrenal failure due to residual adrenal reserve.
- If clinical suspicion of adrenal insufficiency persists despite an initial satisfactory SST, it is important to recognise the need for re-assessment of cortisol levels.
- Insulin tolerance test remains a useful tool in the investigation of secondary adrenal insufficiency.
- Levothyroxine replacement may accelerate the metabolism of cortisol in cases of concurrent hypothyroidism and hypoadrenalism, and therefore, glucocorticoid replacement must precede levothyroxine treatment, to avoid adrenal crisis.
- The prevalence of immunotherapy-related endocrinopathies is likely to increase with increasing use of ICI and it is crucial that clinicians are alert to their subtle symptoms.

## Background

Immune checkpoint inhibitors (ICI) have revolutionised the management of malignancies, including melanoma, non-small cell lung cancer and renal cell carcinoma. Secondary adrenal insufficiency in the context of ICI use has been reported due to both complete pituitary gland inflammation (hypophysitis) and isolated adrenocorticotropic hormone (ACTH) deficiency. Primary adrenal insufficiency is much less commonly reported as it represents a much rarer phenomenon (Joshi *et al.* 2016). Programmed cell death 1 ligand 1 (PD-L1) inhibitors have been linked to the cases of both primary and secondary adrenal insufficiency due to adrenalitis and isolated ACTH deficiency, respectively (Joshi *et al.* 2016, Iglesias *et al.* 2021). Therefore, careful monitoring of pituitary function and especially cortisol levels is essential during immunotherapy.

The Society for Endocrinology (SfE) and the French Endocrine Society have developed guidelines for the investigation of primary and secondary adrenal insufficiency for ICI-induced endocrinopathies, but their recommendations regarding dynamic testing remain ambiguous (Castinetti *et al.* 2019). Specifically, the SfE suggests a 250 μg short synacthen test (SST), if early morning cortisol is between 138 and 500 nmol/L, to diagnose ‘latent’ adrenal insufficiency. SSTs have a high sensitivity and specificity for assessing the hypothalamic–pituitary–adrenal (HPA) axis, without the contraindications, risks and higher cost associated with the gold standard insulin tolerance test (ITT) (Agha *et al.* 2006). The SfE guidelines highlight the need for careful interpretation in cases where ACTH is low, due to a potentially falsely reassuring result in the early stages of pituitary disease.

In this case report, we demonstrate the challenges in monitoring pituitary function during immunotherapy and the pitfalls of interpreting SST results, as illustrated in the case of a patient with metastatic melanoma receiving immunotherapy who, after developing potential symptoms of hypoadrenalism, had two normal SSTs before being diagnosed with secondary adrenal insufficiency. This case highlights the difficulty of using different screening methods for hypocortisolaemia.

## Case presentation

A 53-year-old Caucasian man with metastatic stage 4 melanoma receiving ipilimumab and nivolumab combination immunotherapy attended the Endocrine clinic for a ‘borderline’ low cortisol level of 140 nmol/L (reference range, 119–618 nmol/L) taken in the afternoon. Following three cycles of immunotherapy with no significant side effects, he developed immunotherapy-related thyroiditis with an elevated thyroid-stimulating hormone (TSH) and a low free T4 (FT4) (Table 1). A random cortisol level immediately before the fourth cycle of immunotherapy showed slight improvement (Table 1) but remained below 200 nmol/L. Therefore, an urgent SST using ACTH 1–24 of 250 μg IM was arranged to rule out cortisol deficiency. The patient’s only other medications were amlodipine, bisoprolol and ramipril; there was no history of exogenous steroid or opiate use. The SST demonstrated normal baseline, post-synacthen serum and salivary cortisol levels. A pituitary hormone profile was also normal (Table 1). Only after the inconsistent results with adrenal insufficiency, levothyroxine of 50 μg was commenced once daily.

**Table 1:** Serum blood tests performed during the course of the patient’s treatment and investigations. ACTH levels remained inappropriately normal despite the undetectable cortisol level after the second SST. TFTs gradually improved with dose titration of levothyroxine. The patient had demonstrable ACTH deficiency, primary hypothyroidism and hypogonadotrophic hypogonadism.

	Test	At presentation		First SST (at 10:00 h)	Second SST (at 10:55 h)	3 weeks after second SST (13:40 h)
				3 days after presentation	4 weeks after presentation	7 weeks after presentation
		Post-3 cycles nivolumab			Post-4 cycles nivolumab	Post-5 cycles nivolumab
HPA axis	Cortisol (ref 119–618 nmol/L)	140 (at 15:00 h)	187 (next day at 13:14 h)	331 (baseline)	187 (baseline)	<50
	ACTH (ref 0–46 ng)			30		27
Thyroid function tests	TSH (ref 0.55–4.78 mU/L)	49.3			0.95	1.63
	Free T4 (ref 10–22 pmol/L)	2.2			5.2	8.7
Thyroid antibodies	Anti-TPO antibodies (0–24 IU/mL)	10				
Pituitary screen	LH (IU/L)			2	<1	4
	FSH (IU/L)			3	2	2
	IGF-1 (ref 56–197 ng/mL)			153		176
	Prolactin (mU/L)			136		
	Testosterone (ref 8.4–28.7 mol/L)			12.1	<0.5	20.8
Other adrenal bloods	Renin (0.3–2.2 nmol/L/h)					0.6
	Aldosterone (0–630 pmol/L)					149

Three weeks after the SST, the patient attended his local Emergency Department complaining of headache, fatigue, palpitations, abdominal pain and weakness. His chest x-ray showed right lower lobe consolidation and he was treated with oral antibiotics for community-acquired pneumonia. One week post discharge, he reported persisting headaches, dizziness, progressive fatigue and general malaise but no visual disturbances. These symptoms again raised concerns for adrenal insufficiency due to hypophysitis, despite the recent reassuring SST and pituitary function tests (Tables 1 and 2). His SST was repeated and was again normal. Baseline cortisol levels were taken mid-morning and therefore were difficult to interpret but were lower compared to previously. Repeat testosterone levels were undetectable, with inappropriately low-normal gonadotrophins, but as these were only recently normal, a repeat test was indicated before starting testosterone replacement. It was posited that his symptoms of malaise were related to inadequate levothyroxine replacement, given a low FT4 (Table 1) and his dose was increased.

**Table 2:** Short synacthen test results demonstrating satisfactory serum cortisol responses to synacthen. Salivary cortisol levels were performed with the first SST in case binding proteins were disproportionately affecting the results; however, these were also within normal ranges.

	First SST (10:00 h)	Second SST (10:55 h)
	3 days after presentation	4 weeks after presentation
Baseline	331 nmol/L	187 nmol/L
Cortisol at 30 mins	707 nmol/L	556 nmol/L
Cortisol at 60 mins	836 nmol/L	706 nmol/L

**Figure 1:**
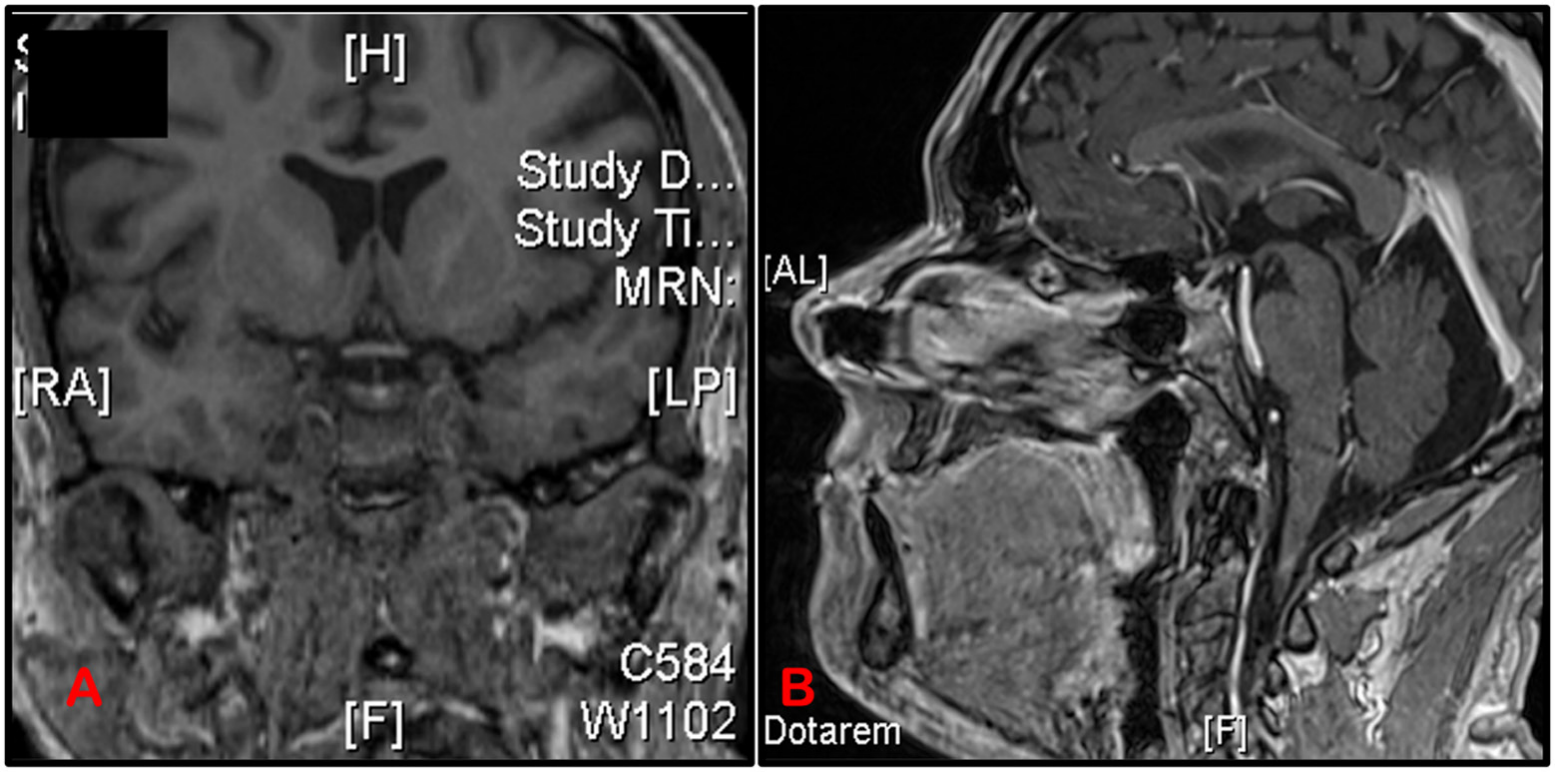
Coronal and sagittal T1 MRI sequences with contrast showing an unremarkable pituitary fossa, with no masses or pituitary enlargement to suggest hypophysitis. The pituitary stalk and hypophyseal region were also unremarkable. However, it is important to note that this MRI was performed 3 months following the initial SST and early inflammatory changes might have resolved by the time of the scan.

Three weeks after the second normal SST, the fatigue persisted. Subsequently, an undetectable cortisol level and inappropriately normal ACTH were noted when we would have expected the undetectable cortisol to trigger an elevated ACTH if this represented primary adrenal insufficiency. Considering his headache and biochemical results, we diagnosed ICI-induced hypophysitis causing ACTH deficiency and therefore secondary adrenal insufficiency. The interpretation of TFTs is hindered by levothyroxine replacement and his other pituitary functions had normalised by this time. The patient’s MRI pituitary was normal (Fig. 1).

In ACTH deficiency with undetectable cortisol, the expectation was for the serum ACTH level to be much lower than it was, despite our interpretation of it being inappropriately normal. Our laboratory uses a Siemens Immulite 2000 analyser^©^ and consequently, we reassessed the ACTH using an alternative analyser (Roche Cobas^©^). We measured pre-hydrocortisone-paired ACTH and cortisol readings on a separate occasion. In our laboratory, with corresponding cortisol of <50 nmol/L, the ACTH measurement was 42 ng/L (0–46), while the corresponding ACTH on the Roche Cobas platform was <3 ng/L (7.2–63.3).

## Investigation

CTs of his thorax, abdomen and pelvis with contrast approximately 2 weeks before and 4 months after the initial SST showed no evidence of metastatic disease in the abdominal or pelvic organs.

## Treatment

The patient was initially commenced on glucocorticoid replacement consisting of hydrocortisone 20 mg in the morning and 10 mg at lunchtime, and his dose of levothyroxine was further titrated to achieve clinical and biochemical euthyroidism.

## Outcome and follow-up

Seven weeks after the initiation of glucocorticoid replacement and optimisation of levothyroxine dose, most of the patient’s symptoms had resolved; however, he still described waning energy levels towards the end of the day, and hence, the timing of his hydrocortisone doses was adjusted.

## Discussion

This case poses two possibilities for this presentation: the most likely being that a normal stimulated cortisol level (SST) is possible in early stages of ACTH deficiency and hypophysitis where the zona fasciculata has not yet atrophied from lack of ACTH stimulation. We believe there was progression of his ACTH deficiency as the original symptomatology preceded the obvious biochemical abnormality and furthermore that although there was a subtle downwards trend in cortisol levels, the peak cortisol response offered false reassurance. Alternatively, the patient may only have developed ACTH deficiency when it was biochemically diagnosed as the temporal relationship to the number of cycles of immunotherapy and the onset of his abnormalities were consistent with the typical accepted timeframe for pituitary abnormality (after three cycles). Importantly, the baseline cortisol at the time of the second SST declined, along with a low-normal TSH and low FT4, which could indicate evolving hypophysitis and favour the former. The normal SST, while reliable in ruling out primary adrenal insufficiency in cases of adrenalitis, may lack sensitivity in early secondary adrenal insufficiency, as in patients with recent pituitary surgery, where 6 week post-opeartive pituitary function monitoring is recommended (Prete *et al.* 2017). With isolated ACTH deficiency, a normal MRI pituitary cannot completely exclude hypophysitis, especially using a delayed scan (Fig. 1).

ACTH serves as a key discriminator between primary and secondary adrenal insufficiency. Potential heterophile antibody interference and lack of standardisation can lead to misdiagnosis of the underlying pathology (El-Farhan *et al.* 2017, Donegan *et al.* 2019). When there is mismatch between the clinical picture and the laboratory results, assay interference should be considered, even in ICI-induced endocrinopathies. The ACTH, while not raised initially, was not low especially when considering the patient had an undetectable cortisol. We concluded that there was false elevation of the ACTH, which was confirmed using an alternative analyser (Roche) and substantiates previous reports in the literature emphasising assay interference (Donegan *et al.* 2019).

Although ITTs remain the gold standard for assessment of ACTH and cortisol reserves, our patient was insufficiently stable for an ITT. A trial of hydrocortisone upon presentation with reinterrogation of the HPA axis after 6 weeks could have been a safer approach despite adding a confounder of glucocorticoid use. Indeed, the role of ITTs in non-urgent situations remains controversial, given the risks of the test, particularly in an older population with underlying malignancy and comorbidities, where the safety of ITT has not been previously assessed. The use of glucagon stimulation test, which has a better safety profile compared to ITT, has not been reported in the literature on ICI-induced adrenal insufficiency and its role remains to be further explored (Berg *et al.* 2010). Our case supports the available guidelines which do not strongly emphasise the use of dynamic function tests and focus on the diagnostic merits of isolated cortisol measurements due to the potential speed of onset and evolution of ICI-related pituitary dysfunction.

Previous studies have shown that hypothyroidism decreases the metabolism of cortisol (Iranmanesh *et al.* 1990). Both primary and secondary adrenal insufficiency may be associated with autoimmune thyroid disease as part of an undiagnosed polyglandular syndrome (Iranmanesh *et al.* 1990, Agha *et al.* 2006, El-Farhan *et al.* 2017). In patients with newly diagnosed autoimmune thyroid conditions, concurrent hypocortisolaemia is possible and newly commenced levothyroxine could potentially accelerate the metabolism of remaining cortisol reserves leading to hypoadrenal crises (Yamamoto *et al.* 2015). In this case, the rapid development of new symptoms following the commencement of levothyroxine replacement therapy, coupled with the significant drop in baseline cortisol levels after levothyroxine titration, implies that this may have been a contributory factor. However, we cannot conclude this with certainty.

In summary, with the increasing use of ICI, cases like these highlight how challenging the clinical presentation and follow-up of patients with ICI-related endocrinopathies/pituitary abnormalities can be. The frequently concurrent bias from exogenous factors such as glucocorticoids complicates diagnosis. An awareness of these pitfalls benefits oncologists and endocrinologists alike and enhances the speed and accuracy of diagnosis for ICI-related HPA abnormalities.

## Declaration of interest

The authors declare that there is no conflict of interest that could be perceived as prejudicing the impartiality of this case report.

## Funding

This work did not receive any specific grant from any funding agency in the public, commercial or not-for-profit sector.

## Patient consent

Written informed consent was obtained from the patient on the 19 April 2021.

## Author contribution statement

Yixi Bi conceived the study, analysed the results and wrote the manuscript. Safwaan Adam conceived the study, contributed to the manuscript and is the endocrinologist responsible for the patient. Viktoria Chatzimavridou contributed to the writing and editing of the manuscript. Paul Lorigan contributed to the writing of the manuscript and is the oncologist responsible for the patient. Yinglai Huang contributed to the writing of the manuscript.
